# Current Modalities for Low Vision Rehabilitation

**DOI:** 10.7759/cureus.16561

**Published:** 2021-07-22

**Authors:** Richa Agarwal, Alka Tripathi

**Affiliations:** 1 Ophthalmology, All India Institute of Medical Sciences, Gorakhpur, IND

**Keywords:** low vision, low vision aids, vision rehabilitation, telescopes, retinal prosthesis

## Abstract

Visual rehabilitation is an effective method for increasing the quality of life among individuals with low vision or blindness due to untreatable causes. Low vision rehabilitation aims for patients to use their residual vision effectively and efficiently to enable them to live independent and productive lives. Low vision rehabilitation includes assessment of residual visual functions, prescription of rehabilitation aids, and training in the use of devices. A multidisciplinary approach and coordinated effort are necessary to take advantage of new scientific advances and achieve optimal results for the patient. This article aims to review the various aids and methods available for low vision rehabilitation and also discusses technology advances that can enhance the visual functioning of individuals who are visually impaired.

## Introduction and background

According to the World Health Organization (WHO), low vision is defined as visual acuity of 3/60 to less than 6/18 in the better eye after best possible correction and visual field <20 degrees from the point of fixation. Functionally, low vision is a level of vision that prevents a person from carrying out their day-to-day activities. Blindness is defined as visual acuity of less than 3/60 and visual field less than 10° [1]. According to the WHO-International Agency for the Prevention of Blindness (IAPB) vision report 2019, 2.2 billion people have a vision impairment or blindness and 1 billion have a vision impairment that could have been prevented or has yet to be addressed [2].

Visual impairment can be either central or peripheral vision loss. The type of rehabilitation varies depending on visual acuity, age, type of visual disability, and individual expectations. The approach to a patient who has central vision loss is different from one who has tunnel vision. Low vision rehabilitation aims for patients to live independent and productive lives by using their residual vision efficiently. It not only includes recommending low vision aids but also training for using these devices and the rehabilitation process. Rehabilitation is a collaborative effort of vocational therapists, social workers, and psychologists, led by an ophthalmologist. This article aims to review the various aids and methods available for low vision rehabilitation and also discusses technological advancement in the field.

## Review

Low vision devices are basically of four types: optical devices, incorporating lenses that achieve optical magnification; non-optical devices, or supplementary devices; electronic devices, using electronic/optical techniques; and complex devices using advanced technologies.

Optical devices

Optics of Low Vision Devices

Low vision devices (LVDs) are those which help people to use their sight to better advantage. The basic principle behind these LVDs is to alter the environment perception through: BBB - bigger, brighter and blacker; and CCC - closer, color and contrast. The basic principle of all low vision optical devices is to magnify. They make use of either or a combination of these types of magnifications: Relative Size: enlargement of the size of the object e.g. large print textbooks; Relative Distance Magnification: moving the object of regard close to the eye so as to subtend a larger image on retina; Angular Magnification: apparent change of size of the object, produced by magnifier or telescopic systems; Electro-optical Magnification: electronic magnification devices and computer software are used to enlarge the objects and also increase the contrast. Illumination remains the most important factor in improving visual function. On adjusting illumination, 90% of patients with vision loss showed improvement in visual acuity [3].

Microscopes (high plus reading glasses)

**Figure 1:**
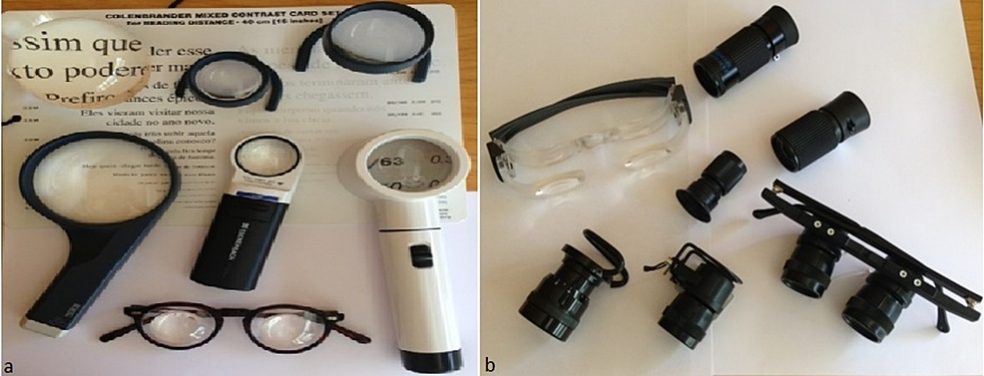
Optical devices (a) Microscopes and various types of magnifiers: hand-held, stand and dome magnifiers (b) Telescopes: Hand-held, spectacle mounted and clip-on type [5]

Glasses with power higher than +3.50Dsph are called high plus add lens (Figure 1a). The higher the power, the closer becomes the reading distance. This is calculated according to Kestenbaum’s rule, the “reciprocal of distance vision”. For example, if the patient has Snellen’s visual acuity of 20/100, the near add is 100/20, which is equal to 5 Dioptres and the near reading distance is 1/5=20cm. This gives the initial add which is gradually increased allowing the patient to read comfortably a text size of 1 M. The magnification produced by these glasses is 1/4th of the power of the lens [4]. They can be full field type, half-frame type or base prism type. Base prism spectacle magnifiers provide convergence of vision as the object has to be viewed from very close by both eyes simultaneously and helps in reducing eye strain.

Magnifiers

Magnifiers can be used along with near vision spectacles. They have a longer working distance as compared to spectacles. However, the greater the distance, the smaller will be the visual field. Magnifiers are available as hand-held, stand, illuminated, fibre-optic, and dome/bar magnifiers (Figure 1a). Hand-held magnifiers are portable, have longer working distances, and are inexpensive. It is helpful in patients with eccentric viewing. However, they have limited field of view and must be held steady at a fixed working distance. Stand magnifiers provide both angular magnification and relative distance magnification. They can be fixed focus, focusable, with or without illumination and rest on a rigid mount. They are technically simpler to use and are a choice for patients with hand tremors, paralysis, arthritis, or poor hand-eye coordination. Dome magnifiers are solid stand magnifiers that look like paperweights. They are placed directly on the text to be read and have excellent light-gathering properties. Bar magnifiers are half-cylinder lenses used for the limited purpose of reading one line at a time. Fresnel prisms use a convex lens effect that is obtained in a thin sheet of acrylic by cutting progressive prismatic grooves concentrically. They are very handy, lightweight and low cost.

Telescopes

Telescopic systems magnify the apparent size of distant objects; they work on the principle of angular magnification. They can be prescribed for near, intermediate and distant tasks. Field of view decreases with magnification and it also varies with design of the telescope. Telescopes are either Keplerian or Galilean or depending on their optical principles. The Keplerian telescope has two convex lenses giving an inverted image which is corrected with prisms, while the Galilean telescope has a convex objective lens and a concave eyepiece lens which gives an erect image. A Galilean telescope is lighter than the Keplerian type, thus the first-choice in children. As a reverse telescope, it provides a wider visual field and can be used in cases of peripheral field loss. Keplerian telescopes have better optical quality and greater visual field but are also expensive. Using telescopes requires good coordination and training. Due to difficulty in using the devices, expense and cosmetic considerations, telescopes are not usually accepted.

Telescopes can be hand-held, clip-on or spectacle-mounted, and can be prescribed monocularly or binocularly (Figure 1b). A monocular telescope is given when the visual acuity between the two eyes is different. Telescopes can be fixed focus, focusable telescope, or autofocus. A focusable telescope can be used for near, far and intermediate distance.

A bioptic telescope is attached to the top of the spectacles, and by just tilting the head, patient can switch between the magnified vision and regular vision. Autofocus telescopes use a motorized focusing system to focus objects at different distances [6]. The ‘in-the-spectacle-lens’ telescopes having a wide-field Keplerian telescope smoothens patient’s orientation and navigation by using the regular and magnified view of the viewing area simultaneously [7].

Peli’s field expansion prisms

Patients with central visual loss benefit from magnification but in those with retinitis pigmentosa (RP), who have peripheral vision loss (PVL), magnification may reduce their existing vision. Such patients may benefit from reverse telescopes that expand the visual field. A two-fold increase in visual field can be achieved by 0.5X telescope but with reduced visual acuity, hence not preferred. Field expansion prisms are one such method that can be used in patients with PVL.

In patients with homonymous hemianopsia, Peli’s field expansion prisms are used with its base towards the side of the field defect, expanding the patient’s visual field in that direction. These prisms are placed on the posterior surface of the spectacle lens in the upper and lower quadrant leaving a central opening of about 12 mm. In patients with tunnel vision, as in RP and choroideremia, a Trifield prism is used. These are placed on one eye, with base-in in the nasal quadrant and base-out in the temporal quadrant of the spectacle lens, providing peripheral vision and the other eye has central vision. As a result, visual field expansion occurs in all directions. The prisms are coloured to reduce double vision and confusion [8]. The prisms are permanently attached to the lens only after the patient is adapted to the field expansion after training exercises [9].

Telescopic contact lenses

A telescopic contact lens is a high-powered minus lens, which when used along with a high-powered convex lens spectacle provides a magnification of up to two times and an enlarged visual field. It is cosmetically more acceptable than a spectacle-mounted telescope [10]. For an upright image, the Galilean design is incorporated in the telescopic contact lens. Since the magnification is limited, these are suitable for patients with mild-moderate visual impairment.

Tremblay et al. in 2013 designed a telescopic contact lens that allowed shift from normal to magnified vision using 3D glasses and electrical polarization [11]. It was a 1.6 mm-thick scleral contact lenses with poor corneal oxygenation making it difficult for long term use [12]. Another such type of scleral telescopic contact lens in which shift between normal and magnified vision is achieved by blinking, uses battery-operated LCD glasses [13].

Telescopic intraocular lenses

**Figure 2:**
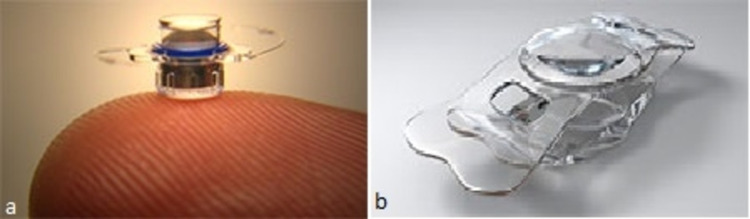
Telescopic intraocular lenses (IOLs). (a) Implantable miniature telescope [14] (b) iolAMD [15]

Telescopic intraocular lenses (IOLs) are used in patients with age-related macular degeneration (AMD), to provide magnification via surgical methods. Till now, seven types of such IOLs have been used in AMD but only short-term results are available and none of them have proven to be ideal. These include the implantable miniature telescope (IMT) (VisionCare Inc., Saratoga, CA, USA) (Figure 2a), IOL-VIP system (Soleko, Pontecorvo, Italy), Lipshitz macular implant (LMI) (Optolight Vision Technology, Herzlia, Israel), sulcus-implanted Lipshitz macular implant (LMI-SI) (OriLens; Optolight Vision Technology), Scharioth macula lens (Medicontur Ltd., Zsámbék, Hungary), iolAMD (London Eye Hospital Pharma, London, UK) (Figure 2b), and Fresnel prism intraocular lens (P-Flex; Rayner Intraocular Lenses Ltd., Worthing, UK). The magnification power of the lenses are as follows: 2.5X with the IMT, 2.5X with the LMI, 1.2X with the iolAMD lens, 1.3X with the IOL-VIP system, and 1X in the Fresnel prism intraocular lens.

The Galilean-type telescope is the most common approach, used in the IOL-VIP System, IMT lenses, and iolAMD. A positive lens is implanted in the posterior chamber in the IOL-VIP System [16]. IMT lens requires a long tube to be implanted through a large corneal incision [14]. The iolAMD causes a shift of the retinal image from a damaged central retinal area by decentration of one of the IOLs [15]. Another telescope approach in LMI and LMI-SI is based on a Cassegrain configuration, which uses mirrors instead of lenses. It can provide high magnification, but has higher manufacturing costs. Additionally, the use of small mirrors might generate the risk of glare effects, due to diffraction and ghost reflections in the elements [17,18]. The Fresnel Prism IOLs also displaces the retinal image from a damaged central retinal area to a peripheral healthier retinal area with no magnification [19]. Diffraction and scattering of light at the edges of the Fresnel zone is the problem faced with these IOLs. The Scharioth macula lens is based on magnification at closer distances in a range of 10 to 15 cm from the eye. The closer the object to the eye, the higher the magnification and for this it has a +10 D central area in the lens for accommodation. It provides no distance vision magnification [20]. There are also difficulties in fundus imaging after implantation of the telescopic IOLS [21].

Electro-optical systems

The primary electro-optical device is a standard closed circuit television (CCTV) system. It has a camera to capture and display enlarged images or text on a computer screen or TV. Magnification, contrast, brightness, change of polarity from black to white and other features such as voice command can also be controlled in these devices [22], overcoming the problems of magnifying systems, like short working distance, reduced visual field, reduced contrast, and illumination. While most CCTVs are desktop units, portable (hand-held and head mounted) units are also available. Mouse magnifiers are like a computer mouse having a camera that is moved over the text to be read. They are portable and can be connected to personal computers. Magnification, focussing and reverse contrast can be controlled in these devices but the viewing area is limited.

Computer Technology

Computers play an important role in low vision rehabilitation process as internet is the main source of information nowadays. Adaptive computer hardware and software offer accessibility to electronic and printed materials to patients with severe visual loss. Adaptive hardware such as large monitor screens, modified keyboards, and attached scanners makes it feasible to use the software. Software programs allow either large magnification or translation to auditory perception. These devices are also used by individuals with normal vision, so they don’t stigmatize the ones with visual impairment. JAWS screen reading software for the blind converts a normal PC into a talking computer. Connect outloud internet and e-mail allow user to access internet and email through speech and Braille output. MAGic 8.0 is screen magnification software which reads the information aloud as users type it, or move the mouse over it. Various other softwares are available like Zoom and VoiceOver app, ZoomText, etc. Many software applications are also available for smart phones which can make them work like a hand-held magnifier e.g., Brighter and Bigger, and Better Vision apps.

Non-optical systems

Non-optical systems increase the patient’s residual visual function or use signals that stimulate one of the other senses. Illumination, large-print books, increased contrast, typoscope, reading stands, and sunglasses or spectacles with filtering lenses to reduce glare can be used alone or in conjunction with optical systems in patients with low vision.

Independent living devices

Devices for daily living, like colour identifiers, talking glucometers, talking scales, talking compasses, Braille alphabet, audio books, voice recording devices and a variety of other devices are now available for assistance of visually impaired.

Driving

Talking Global Positioning System (GPS) devices are available which should be recommended to drivers with visual impairment. Distraction and time spent in looking for road signs are minimised using this device. Lane alert warnings, adaptive cruise control, cars that park themselves and continued technological advances in automobile industry will make driving safe for all drivers.

Walking

**Figure 3:**
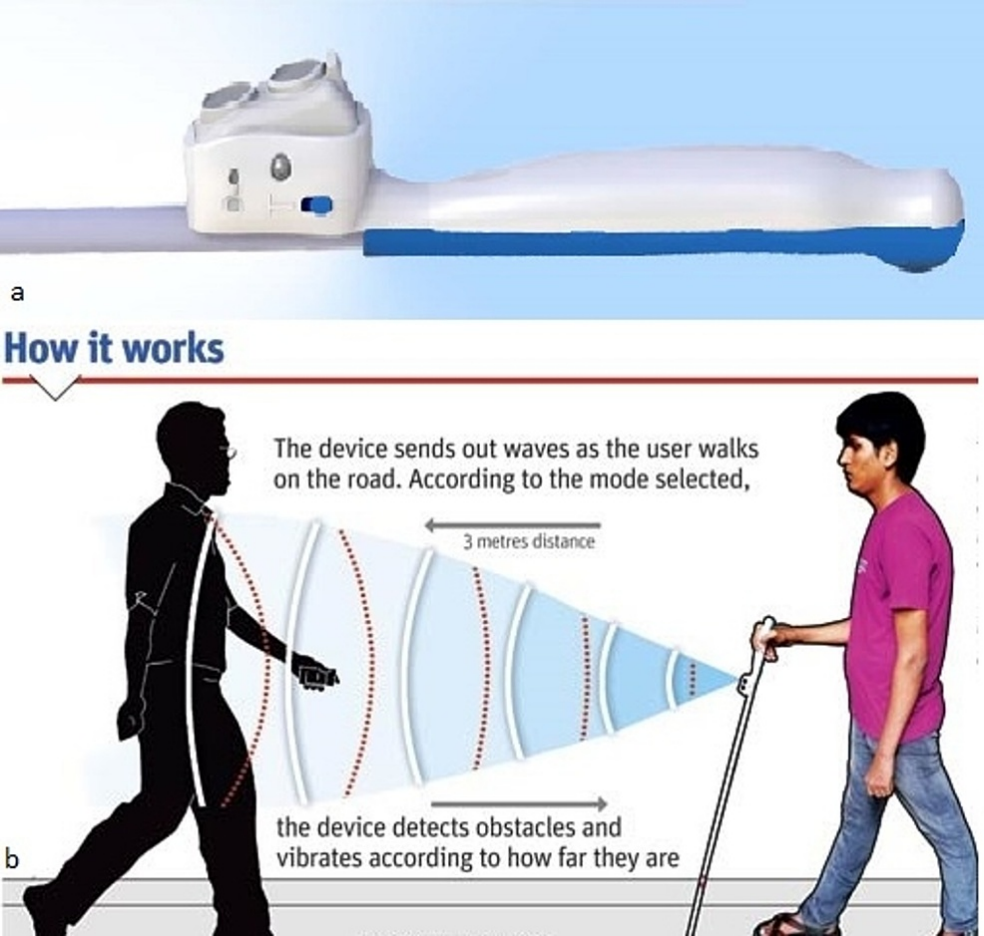
Smart cane Smart cane [24] (Graphic courtesy: Amit Bandre)

Smart Cane device (Assistech, IIT, New Delhi, India) is an electronic walking aid that fits on the top of the white cane (Figure 3). It overcomes the limitations of white cane, by detecting knee above and hanging obstacles and the detection distance is increased from 0.5 meters to 3 meters. It informs about the presence of objects before actually touching the object with the cane and thus helps in preventing unwanted contact. It uses ultrasound technology to detect obstacles in its path and generates different form vibratory patterns. These vibratory patterns convey the information about the distance and allow the user to identify the objects from distance. With simple training and orientation, the device can benefit any visually impaired person using the white cane regularly [23].

Microperimetry

Microperimeters map the pattern of a patient’s response at various retinal points to the light stimuli and superimpose that data on the fundus image captured by fundus photography or scanning laser ophthalmoscopy to exactly identify areas of preserved or impaired function. Accurate testing is ensured by eye-tracking technology which corrects for eye movement. It can identify scotomata that they do not correlate with fundus appearance and can give the correlation of residual vision and functional vision [25,26].

Microperimetry guides rehabilitation efforts and help in training patients to identify and to make maximum use of the areas of best vision. In patients with central vision loss, eccentric fixation develops in healthier retinal regions, called the preferred retinal locus (PRL). Most patients being unaware of their PRL, move their heads around to locate it. This can be detected by microperimetry and the patient can be taught to find the PRL quickly and use it efficiently. Prism glasses can be used to redirect images toward the PRL. Microperimetry has a biofeedback feature which allow to relocate the PRL to an area of higher retinal sensitivity when it is not in an appropriate place, with PRL shifting exercises (trained retinal locus, TRL) [27]. Indications of microperimetry include low vision due to AMD, RP, Stargardt disease, diabetic retinopathy, and glaucoma [28].

Retinal prosthesis

Retinal prostheses are implantable electronic devices that stimulate visual sensation within the eyes of individuals with retinal diseases such as AMD and RP, where the optic nerve is normal. Due to its accessibility and neuronal architecture, the retina is the easiest target for treating outer retinal disease. Retinal prostheses are categorized based on electrical impulse delivery mechanism and where in the eye the device is to be placed, which can be subretinal or epiretinal. The advantage of epiretinal device is that it can be easily implanted with minimal risk but subretinal devices have the advantage of using middle retinal layer formed by bipolar, horizontal and amacrine cells [29]. An electrical impulse is delivered by either micro-electrode arrays or micro-photodiode arrays. The former has a camera, processing unit, and a transformer which convert the light to electric impulses by a micro-electrode array while the latter has an array of photodiodes which on receiving the light directly generate electrical current, so it does not need a camera, processor, or transformer [29]. On the other hand, micro-electrode arrays are able to generate larger electrical current and use various light processing algorithms to highlight edges, contrast and other features. Two such devices, Argus II retinal prosthesis (Second Sight Medical Products, Sylmar, CA, USA) and Retina Implant Alpha IMS (Retina Implant AG, Reutlingen, Germany), are CE Mark approved and are currently being implanted to address blindness from advanced RP.

Argus II Retinal Prosthesis

**Figure 4:**
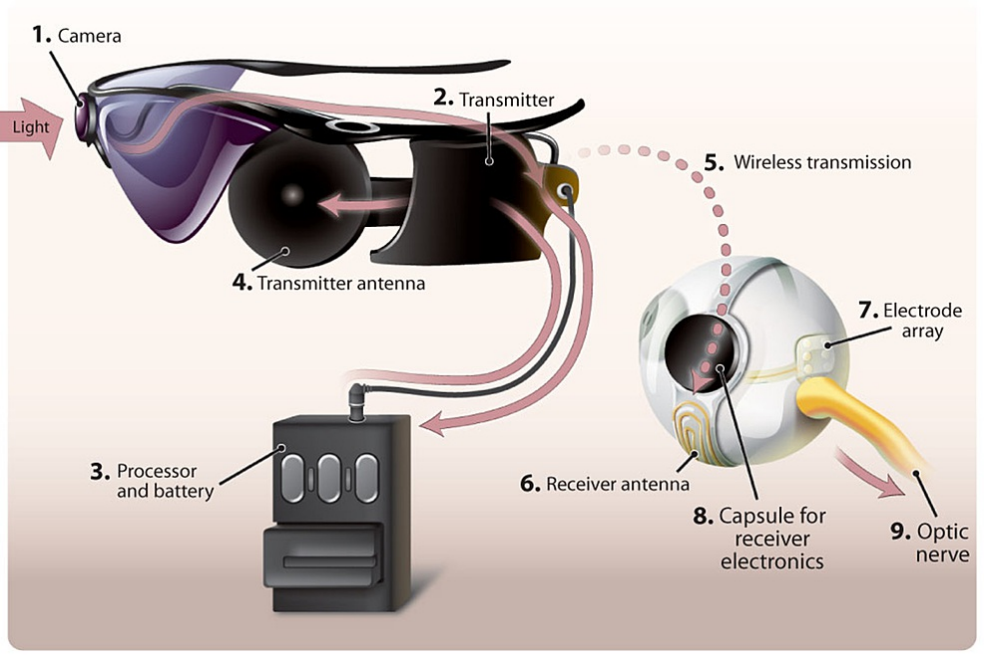
Argus II. External components: camera mounted on glasses, processor and transmitter. Internal components: receiver coil, electronic capsule and an array of electrodes [33]

This is the first prosthesis approved by the Food and Drug Administration. The Argus II is a micro-electrode epiretinal prosthesis, delivers electrical impulses to the retinal ganglion cells which produce phosphenes, the light spots [30]. It consists of three internal components and three external components [31] (Figure 4). Patients require training and rehabilitative support to adapt to this system of artificial vision once it is implanted. Studies have shown improvement in visual function tests in patients implanted with Argus® II in terms of ability to recognize and discriminate forms, detect motion, and navigate [32]. The Argus® II is indicated in patients with severe RP older than 25 years of age, have only or no light perception in both eyes, with a previous history of some useful vision. It is implanted in one eye, usually the worse-seeing eye. Patient's willingness for the recommended post-implant follow-up, training, and visual rehabilitation is a very important prerequisite.

Retina Implant Alpha-IMS

This is a micro-photodiode, subretinal prosthesis, implanted within the layer of degenerated photoreceptor cells. Electrical signals generated by the device stimulate bipolar cells in the middle retinal layers which carry the impulses to retinal ganglion cells. It’s a 1,600-pixel microphotodiode array relying on light to stimulate the optic nerve via remaining RPE cells [34]. The implant includes a handheld device that transmits energy via magnetic induction to a coil implanted under the skin behind the ear. It is indicated in patients with degenerative outer retinal disease.

BrainPort

BrainPort (Wicab, Inc., Middleton, WI, USA) provides artificial vision to patients who have previously experienced some sort of vision. It consists of a camera mounted on glasses, which captures and sends the image to a handheld remote-control unit where the image is converted into a black and white low-resolution photo. This is then transmitted to an array of electrodes placed on tongue, which sends sensory information to the brain. These impulses are then interpreted as visual signals and are redirected to the visual cortex, allowing the person to "see" [35,36].

Stem cells

Stem cells can replace diseased retinal cells with new retinal cells because of the ability of self-renewal and differentiation into mature cells. It can be used for degenerative diseases of the retina such as RP, AMD and Stargardt macular dystrophy, with the aim of slowing disease progression and preserving the visual field. Adult mesenchymal stem cells from adipose tissue and bone marrow are most commonly used in low vision patients [37]. It is given as a subretinal injection with total vitrectomy in the worse eye of patients above 18 years of age. However, uncertainties still exist regarding type of stem cells, dose and route of administration. In a study by Oner et al. [38], only one out of 11 RP patients showed improvement in visual acuity, visual field and electroretinogram results. They also noted ocular complications related to the procedure. The complications can be minimized by suprachoroidal administration reported in another study [39].

**Table 1:** Studies using MSCs for retinal degenerative diseases. BM-MSC: bone marrow-derived mesenchymal stem cells; A-MSC: adipose tissue-derived mesenchymal stem cells; AMD: age-related macular degeneration; RP: retinitis pigmentosa

Retinal Disease	Stem cells/ Route of administration	Result	Study
RP, cone-rod dystrophy	Autologous BM-MSC/ intravitreal	Phase 1, no severe safety issues	Siqueira et al. [40]
AMD, RP, retinal vascular occlusion	Autologous BM-MSC/ intravitreal	Phase 1, no severe safety issues	Park et al. [41]
Optic nerve diseases	Autologous BM-MSC/ retrobulbar, subtenons, intravitreal, intravenous	Improvement in visual function	Levy et al. [42]
RP	A-MSC/ subretinal	Minor ocular complicaions, no severe safety issues	Oner at al. [38]
Ischemic optic neuropathy	Autologous BM-MSC/ retrobulbar, subtenons, intravitreal, intravenous	Improvement in visual function	Weiss et al. [43]
RP	Autologous BM-MSC/ retrobulbar, subtenons, intravitreal, intravenous	Improvement in visual function	Weiss et al. [44]
RP	Umbilical cord- derived MSC/ suprachorodial	Improvements in visual acuity, electroretinography and visual field	Kahraman N. S. [45]
RP, inherited retinal dystrophy	Wharton’s jelly-derived MSC/ subtenons	Improvement in visual acuity and in outer retinal thickness	Oumlzmert et al. [46]

Platelet-rich plasma (PRP) therapy

Autologous blood is centrifuged to concentrate platelets and is then injected. The growth factors produced by platelets (NGF, BDNF, BFGF, IL-6) help to maintain the viability of the photoreceptor cells. The aim is to increase visual acuity, expand the visual field, and to slow the progression of disease. In a study by Arslan et al., 49 out of 71 eyes of 48 patients with RP who received PRP injection showed significant improvements in microperimetry and multifocal electroretinogram values with positive visual outcome [47].

Non-invasive brain stimulation

Transcorneal Electrical Stimulation (TES)

TES is a technique whereby electrical stimulation in low-dose is applied to retinal cells given trans-corneally. This causes a release of neurotrophic growth factors helping to protect and prevent further loss of the residual intact retinal cells. It is done once a week for six to eight weeks and can be combined with PRP injection. Bittner and Segeer [48] reported significant improvements in visual acuity, contrast sensitivity, and/or Goldmann visual field test results in four of seven patients in the RP group after six weeks of TES therapy. After follow-up for 29-35 months, three patients showed no regression.

Transcranial Electromagnetic Stimulation (TMS)

TMS uses magnetic stimulation delivered centrally and not applied locally to the retina. The aim is to reduce cell death by stopping the apoptosis cascade and revival of dormant photoreceptors leading to expansion of the visual field. It can also be performed along with PRP injection.

Gene therapy

Gene therapy is a technique that fixes a genetic problem at its source, used for treating autosomal recessive and X-linked diseases. A genome encoding a functional product, carried by vectors like lentiviruses and adenoviruses, is given as a subretinal injection to produce a therapeutic effect. The most studied diseases in terms of gene therapy are Leber’s congenital amaurosis (LCA) and RP [49]. LUXTURNA^TM ^(voretigene neparvovec-rzyl; Spark Therapeutics, Inc., Philadelphia, PA, USA) is the first approved gene therapy for an inherited retinal disorder in the U.S. and also in the European Union. Luxturna delivers a functional copy of the RPE65 gene into the eye [50]. Indications for gene therapy include a significant decrease in vision and the presence of intact retinal cells that can be repaired with gene therapy. For a single disease, the presence of large number of disease-causing genes and their mutations is the hurdle to this method of gene therapy. For instance in RP, more than 160 genes and their different mutations have been identified. Many other gene therapies like RPGR and NSR-RPGR genes in X-linked RP, CNGA3 and CNGB3 genes in achromatopsia, CRISPR/Cas9 gene in LCA 10 and ABCA4 gene in Stargardt disease are under clinical trials [51].

## Conclusions

Visual rehabilitation allows individuals with low vision to lead an independent and improved quality of life. Low vision rehabilitation includes not only prescribing but also training for using the devices. Factors to be considered for deciding the treatment options are diagnosis, age, education level, socio-economic status and patient’s expectations. There are many promising developments in this field but it is also important take care of patient’s living conditions. Taking measures such as sitting students in the front row of the classroom, organizing the home environments in a contrasting and appropriate way, and accentuating steps and handrails will make daily life easier. The article is to present a comprehensive model of visual rehabilitation, summarizing technological advancement in the field and emphasizing the coordinated multidisciplinary approach required in this field. This article should encourage and raise awareness regarding various visual rehabilitative methods for the benefit of all.
